# Predictors of Engagement, Response to Follow Up, and Extent of Alcohol Reduction in Users of a Smartphone App (Drink Less): Secondary Analysis of a Factorial Randomized Controlled Trial

**DOI:** 10.2196/11175

**Published:** 2018-12-14

**Authors:** Claire Garnett, Olga Perski, Ildiko Tombor, Robert West, Susan Michie, Jamie Brown

**Affiliations:** 1 Department of Behavioural Science and Health University College London London United Kingdom; 2 Department of Clinical, Education and Health Psychology University College London London United Kingdom

**Keywords:** smartphone, mobile phone, alcohol drinking, mobile apps, engagement

## Abstract

**Background:**

Digital interventions for alcohol can help achieve reductions in hazardous and harmful alcohol consumption. The Drink Less app was developed using evidence and theory, and a factorial randomized controlled trial (RCT) suggested that 4 of its intervention modules may assist with drinking reduction. However, low engagement is an important barrier to effectiveness, and low response to follow up is a challenge for intervention evaluation. Research is needed to understand what factors influence users’ level of engagement, response to follow up, and extent of alcohol reduction.

**Objective:**

This study aimed to investigate associations between user characteristics, engagement, response to follow up, and extent of alcohol reduction in an app-based intervention, Drink Less.

**Methods:**

This study involved a secondary data analysis of a factorial RCT of the Drink Less app. Participants (N=672) were aged 18 years or older, lived in the United Kingdom, and had an Alcohol Use Disorders Identification Test score >7 (indicative of excessive drinking). Sociodemographic and drinking characteristics were assessed at baseline. Engagement was assessed in the first month of use (number of sessions, time on app, number of days used, and percentage of available screens viewed). Response to follow up and extent of alcohol reduction (change in past week consumption) were measured after 1 month. Associations were assessed using unadjusted and adjusted linear or logistic regression models.

**Results:**

Age (all unstandardized regression coefficients [B] >.02, all *P*<.001) and post-16 educational qualifications (all B>.18, all *P*<.03) were positively associated with all engagement outcomes. Age (odds ratio [OR] 1.04, *P*<.001), educational qualifications (OR 2.11, *P*<.001), and female gender (OR 1.58, *P*=.02) were positively associated with response to follow up. Engagement outcomes predicted response to follow up (all OR>1.02, all *P*<.001) but not the extent of alcohol reduction (all −.14<B<−.06, all *P*>.07). Baseline drinking characteristics were the only variables associated with the extent of alcohol reduction among those followed up (all B>.49, all *P*<.001).

**Conclusions:**

Users of the alcohol reduction app, Drink Less, who were older and had post-16 educational qualifications engaged more and were more likely to respond at 1-month follow up. Higher baseline alcohol consumption predicted a greater extent of alcohol reduction among those followed up but did not predict engagement or response to follow up. Engagement was associated with response to follow up but was not associated with the extent of alcohol reduction, which suggests that the Drink Less app does not have a dose-response effect.

**Trial Registration:**

International Standard Randomised Controlled Trial Number ISRCTN40104069; http://www.isrctn.com/ISRCTN40104069 (Archived by WebCite at http://www.webcitation.org/746HqygIV)

## Introduction

### Background

Excessive alcohol consumption is a priority for public health and has a large economic impact on society because of lost productivity, crime, and health care costs [1-3]. Digital behavior change interventions (DBCIs) focused on alcohol reduction show promise as they can help achieve reductions in hazardous and harmful alcohol consumption [4]; can improve the accessibility of support; have a low incremental cost (once developed); are anonymous, and avoid potential stigma associated with seeking help in person. Smartphone apps (“apps”) have the added advantage of being almost constantly available and, therefore, able to provide support when and where needed. Drink Less is an alcohol-reduction app aimed at those who consume alcohol excessively (defined as an Alcohol Use Disorders Identification Test [AUDIT] score of 8 and above [5]). The Drink Less app was developed systematically and in line with the principles of Open Science and consisted of 1 core module and 5 experimental intervention modules (described in detail elsewhere [6]). A first-phase factorial randomized controlled trial (RCT) suggested that combinations of 4 of the intervention modules assisted short-term drinking reduction; however, it also showed that the app suffered from high rates of attrition [7].

Eysenbach’s law of attrition distinguishes between 2 types of attrition: nonusage and dropout [8]. Nonusage attrition refers to the complete lack of, or low, engagement with a DBCI. Engagement with DBCIs can be defined as “the extent of DBCI use (eg, amount, depth, frequency, duration)” [9] and can be measured through automatic recording of DBCI use. A minimum level of engagement with an intervention is assumed to be necessary for that intervention to have its desired effect, although there is no research on what constitutes the minimum required. However, observed levels of engagement with available DBCIs have often been considered too limited to support behavior changes [10]. The second type of attrition—dropout—refers to participants being lost to follow up. A low rate of response to follow up is a major methodological challenge in intervention evaluation [11] because it reduces statistical power and, therefore, the ability to estimate effectiveness accurately [12]. Trials of DBCIs—especially those involving remote recruitment—appear particularly vulnerable to low response rates to follow up [8,13,14].

The law of attrition also proposes that engagement and response to follow up are positively associated: if users stop engaging with an intervention, then they are unlikely to respond to follow up [8]. This positive association between engagement and response to follow up was found in a systematic review of Web-based health interventions [15] and in each trial arm of an RCT of a Web-based alcohol intervention [16]. Although the relationship may depend upon the intervention and context in which it is being studied, there have been other reports of higher follow-up responses in the control condition if the intervention arm was particularly demanding [17,18]. It may be that users become fatigued in the experimental condition and decide too much time has already been dedicated to the trial.

To improve the likelihood of behavior change and the validity of DBCI trial’s results, research is needed to understand whether certain users are less likely to engage with the intervention, respond to follow up, or change behavior. The identification of predictors of engagement, response to follow up, and behavior change could inform the development of tailored strategies for specific user groups in DBCI trials. The relationship between engagement and response to follow up has not yet been evaluated in an app-based alcohol intervention.

### Predictors of Engagement and Response to Follow Up

Existing literature indicates that being female, older, and better educated predicts higher engagement and greater response to follow up in Web-based alcohol interventions [16,19-21]. Drinking characteristics tend to have an impact on engagement and response to follow up in opposite directions; people at less risk of alcohol harm [20,21] and consuming fewer units a week [16,21,22] are more likely to respond to follow up, though they show lower levels of engagement [16,22-24]. However, some of these studies involved the specific population of students [22-24], and there are also inconsistencies in the evidence when studying problem drinkers [25,26]. Hence, these findings may not be generalizable to the general population, and current evidence regarding user characteristics that may predict engagement and response to follow up in Web-based alcohol interventions is ambiguous. There is also a lack of evidence specifically relating to app-based alcohol interventions, which may differ from the equivalent Web-based intervention in terms of user characteristics. A recent study comparing users of app and Web-based versions of the same Drinks Meter intervention found that app users were younger and had higher levels of alcohol consumption compared with website users [27].

### Predictors of Alcohol Reduction

Drinkers in England who report an attempt to reduce their drinking are more likely to be older, female, of higher socioeconomic status, have high levels of alcohol consumption, and less likely to be white [28]. Attempts and success in those attempts are distinct and likely to be independently predicted, and there is a lack of research on the predictors of successful attempts to reduce drinking, particularly in the context of DBCIs. Understanding which users are less likely to be successful in reducing their drinking when using a DBCI can inform the development of additional strategies to support them and help identify those who are more likely to require face-to-face support. It is also important to understand the relationship between engagement with a DBCI and behavior change and to establish whether there is a threshold level of engagement required to achieve the intended outcomes of the DBCI [29]. However, some studies, conducted across behavioral domains and study settings, have found a positive association between engagement and successful behavior change, suggesting a dose-response relationship may exist [29-32]. There is no research to date on the relationship between engagement and alcohol reduction in app-based interventions. It is unwise to assume that these relationships will be consistent across different behaviors and types of digital interventions; a recent study found different levels of engagement with a digital stress management intervention, depending on whether it was delivered by an app or a website [33].

### This Study

This study investigated the associations between user characteristics, engagement, response to follow up, and extent of alcohol reduction in an app-based intervention, Drink Less, and addressed the following research questions:

1. What associations, if any, exist between user characteristics (sociodemographic and drinking) measured when drinkers register with the app and (a) engagement, (b) likelihood of response to follow up, and (c) extent of alcohol reduction at follow up among those followed up?
2. What associations, if any, exist between engagement and (a) likelihood of response to follow up and (b) extent of alcohol reduction at follow up among those followed up?

## Methods

### Design

The study design is secondary data analysis from a factorial RCT of the Drink Less app between May and August 2016 (reported in full elsewhere [7]). This analysis was not planned before the factorial RCT. Ethical approval was provided by the University College London’s ethics committee (“Optimisation and implementation of interventions to change health-related behaviours” project [CEHP/2013/508]).

### Sample and Recruitment

Participants were eligible if they were: aged 18 years or above; lived in the United Kingdom; had an AUDIT score of 8 or above (indicative of excessive alcohol consumption warranting intervention [5]); confirmed that they were interested in reducing their drinking; provided an email address; and downloaded a *trial version* of the app. People who downloaded the app more than once were removed, with the first case of download retained for the trial. The app was listed in the iTunes store and was promoted through organizations such as Public Health England and Cancer Research UK. The sample size of 672 was prespecified and calculated, so there was more than 80% power to detect a mean change in alcohol consumption of 5 units between the intervention modules.

### Intervention

Drink Less was designed as a stand-alone intervention available to anyone seeking digital support for reducing excessive alcohol consumption. It is centered on a goal-setting module with 5 intervention modules: (1) normative feedback, (2) cognitive bias retraining, (3) self-monitoring and feedback, (4) action planning, and (5) identity change. The app also contains standard features such as the AUDIT questionnaire and feedback on users’ results, the UK drinking guidelines, and links for additional support. Usability testing was conducted during the original app development to understand the user experience and refine the app [34]. Each intervention module existed in 2 versions: *enhanced* (the hypothesized active ingredients for reducing alcohol consumption) and *minimal* (the control). The intervention involved 32 possible options (2^5^: 2 versions of the 5 intervention modules) that users could be randomly allocated to. Users completed the AUDIT questionnaire, sociodemographic assessment, and normative feedback module in a tunneled approach before arriving to the main dashboard. Users were then provided with a stepped guide to aid them in exploring the app, although this was optional and users were free to navigate the app as they wished. Full details on the intervention are reported elsewhere [35], and the app is freely available on the iTunes store [36].

### Procedures

Data collection began on May 18, 2016, and ended on August 28, 2016. On first opening the app, each user was provided with a participant information sheet and asked to provide consent to participate in the trial. Users who consented were asked to complete the AUDIT and a sociodemographic questionnaire, indicate whether they were interested in drinking less alcohol, and provide their email address for follow up. Users were then given their AUDIT score and informed of their *AUDIT risk zone*. At this point, users who met inclusion criteria were randomized to 1 of the 32 experimental conditions in a block randomization method by the app. The follow-up questionnaire consisted of the AUDIT and usability measures and was conducted 1 month (28 days) after first using the app by means of an in-app questionnaire or by a Web-based survey (Qualtrics) that was distributed by email.

### Measures

Engagement was measured as a continuous variable through automatic recording of the extent of DBCI use in terms of amount, depth, frequency, and duration in the 28-day period following registration [9]: number of sessions (ie, frequency of use), a new session was defined as a new screen view after 30 minutes of inactivity [37]; time on app, in minutes (ie, amount); number of days used (ie, duration); and percentage of available screens viewed (ie, depth)—the percentage of unique screens viewed by the user, out of the number of screens available to view (differed depending on treatment group, ranged from 50 to 80).

Response to follow up was a binary (yes or no) measure of completion of the 1-month follow-up questionnaire. The extent of alcohol reduction was measured as the change in past week alcohol consumption (−90 to +90 units) derived from the Alcohol Use Disorders Identification Test-Consumption (AUDIT-C; a brief screening test for alcohol consumption) between time of registration and 1-month follow up.

User characteristic variables were measured at baseline, on first opening the app, and assessed: age (continuous); gender (male or female); employment status (dichotomized into employed vs not employed); ethnicity (dichotomized into white vs not white); education (dichotomized into pre-16 and post-16 educational qualifications); whether they were a current smoker (yes or no); past week alcohol consumption derived from the AUDIT-C (ranging from 0 to 90 units), and full AUDIT score (ranging from 0 to 40).

### Analyses

All analyses were conducted using R version 3.4.0, and the analysis plan was preregistered on the Open Science Framework [38]. Participants with missing data on any variable of interest were excluded from the analyses. The assumptions for a parametric test were assessed (eg, normality of the distribution of residuals), and if these assumptions were not met, the appropriate nonparametric test or transformation (eg, log() transformation for positively skewed data) was used. Descriptive statistics (mean [SD] or median [interquartile range, IQR] to account for a positive skew or n [%], as appropriate) were used to report on the variables included in the analyses (user characteristics, engagement measures, response to follow up, and extent of alcohol reduction).

Generalized linear modeling (linear or logistic, as appropriate) was used to examine the associations between user characteristics (predictor variable) and engagement, response to follow up, or extent of alcohol reduction (outcome variables). Both unadjusted (univariate) and fully adjusted (multivariable) regression models were reported. Treatment group was included in all adjusted analyses as it is a factor relating to the DBCI that may predict engagement [9], response to follow up [16,39], and extent of alcohol reduction [4]. Past week alcohol consumption was not included in this adjusted model because of anticipated high collinearity between past week alcohol consumption and full AUDIT score.

Generalized linear modeling (linear or logistic, as appropriate) was used to examine the associations between engagement (predictor variable) and response to follow up or extent of alcohol reduction (outcome variables). Both unadjusted and fully adjusted (for treatment group and any predictors of the outcome variables) regression models are reported.

Sensitivity analyses were conducted in which the engagement measures were dichotomized into high or low groups and entered in a logistic regression model to see whether the pattern of results differs.

## Results

### User Characteristics, Engagement Measures, Response to Follow Up, and Extent of Alcohol Reduction in Those Followed Up

A total of 672 participants were included. The mean age was 39.2 years; over half were females (377/672, 56.1%); and the majority were employed (581/672, 86.5%), white (640/672, 95.2%), and had post-16 years’ educational qualifications (484/672, 72.0%). About a quarter of participants (165/672, 24.6%) were current smokers and participants consumed a mean of 39.9 units of alcohol in the past week and had a mean AUDIT score of 19.1, indicating excessive alcohol consumption.

Table 1 reports the user characteristics, engagement measures, response to follow up, and, among those who were followed up, extent of alcohol reduction. In the 28 days following registration, participants used the app a median of 5 times and the median number of days the app was used was 4. The median time on the app was 17 minutes 14 seconds, and participants viewed a mean of 39.0% of the available screens. In total, 26.6% of participants (179/672) responded to follow up and of these, 83.2% (149/179) responded through a Web-based survey. There was a mean 14.3 unit reduction in past week alcohol consumption among those participants who responded to follow up.

### Associations Between User Characteristics and Measures of Engagement

Tables 2 and 3 report the linear regression models in which the engagement measures (number of sessions, time spent on the app, number of days used, and percentage of available screens viewed) were regressed onto the user characteristic variables. Overall, 3 engagement measures—number of sessions, time spent on the app, and number of days used—were log-transformed because of the non-normality of residuals.

Age was significantly positively associated with all 4 measures of engagement. Education level was significantly positively associated with all 4 measures of engagement: number of sessions, time spent on the app (only when adjusted for other user characteristics and treatment group), the number of days on which the app was used, and the percentage of available screens viewed. Older users and those with post-16 educational qualifications were more likely to have a greater number of sessions, spend more time on the app, use the app on a greater number of days, and view a larger percentage of available screens.

Gender was significantly associated with the percentage of available screens viewed by the user in both unadjusted and adjusted models; users who were female viewed a greater percentage of screens available to them. No other user characteristics were associated with engagement with the Drink Less app.

### Associations Between User Characteristics and Likelihood of Response to Follow Up

Table 4 reports the unadjusted and adjusted logistic regression models assessing the association between user characteristics and likelihood of responding to follow up. Age, gender, and education were all significantly associated with likelihood of responding to follow up in both unadjusted and adjusted models. Users who were older, female, and had post-16 educational qualifications were more likely to respond to follow up. Current smoking status was significantly associated with the likelihood of responding to follow up in the unadjusted model, but not in the adjusted model. Users who were not current smokers were more likely to respond to follow up when other sociodemographic variables, AUDIT score, and treatment group were not adjusted for.

**Table 1:** User characteristics, engagement, response to follow up, and extent of alcohol reduction (N=672).

User characteristics		Statistics
Age (years), mean (SD)		39.2 (10.9)
**Gender**		
	Female, n (%)	377 (56.1)
**Employment status**		
	Employed, n (%)	581 (86.5)
**Ethnicity**		
	White, n (%)	640 (95.2)
**Education**		
	Post-16 years, n (%)	484 (72.0)
**Current smoker**		
	Yes, n (%)	165 (24.6)
Past week alcohol consumption in units, mean (SD)		39.9 (27.3)
AUDIT^b^ score, mean (SD)		19.1 (6.6)
**Engagement measures (n=672)**		
	Number of sessions, median (IQR^a^)	5 (2-17)
	Time on app in min:s, median (IQR)	17:14 (8:53-37:19)
	Number of days used, median (IQR)	4 (2-13)
	Percentage of available screens viewed, mean (SD)	39.0 (13.3)
**Response to follow up measure (n=672)**		
	Completion of 1-month follow up, n (%)	179 (26.6)
**Extent of alcohol reduction in those followed up (n=179)**		
	Reduction in past week alcohol consumption in units, mean (SD)	14.3 (24.1)

^a^IQR: interquartile range.

^b^AUDIT: Alcohol Use Disorder Identification Test.

**Table 2:** The effect of user characteristics on measures of engagement (number of sessions and time on app).

User characteristics		Sessions, median (IQR^a^)	Unadjusted simple linear regression		Adjusted^b^ multiple regression		Time on app (min), median (IQR)	Unadjusted simple linear regression		Adjusted^b^ multiple regression	
			B^c^ (95% CI)	*P* value	B (95% CI)	*P* value		B (95% CI)	*P* value	B (95% CI)	*P* value
Age (years)		—^d^	.02 (0.02 to 0.03)	<.001	.03 (0.02 to 0.03)	<.001	—	.03 (0.02 to 0.03)	<.001	.03 (0.02 to 0.03)	<.001
**Gender**											
	Male (reference; n=295)	7 (2 to 18)	—	—	—	—	16 (8 to 34)	—	—	—	—
	Female (n=377)	5 (2 to 15)	.14 (−0.05 to 0.33)	.14	.12 (−0.07 to 0.31)	.21	18 (9 to 41)	.13 (−0.02 to 0.29)	.09	.11 (−0.03 to 0.26)	.13
**Employment status**											
	Unemployed (reference; n=91)	5 (1 to 15)	—	—	—	—	15 (9 to 37)	—	—	—	—
	Employed (n=581)	6 (2 to 17)	.23 (−0.04 to 0.51)	.10	.27 (−0.01 to 0.54)	.06	18 (9 to 37)	.06 (−0.16 to 0.28)	.61	.12 (−0.10 to 0.34)	.27
**Ethnicity**											
	White (reference; n=640)	5 (2 to 17)	—	—	—	—	17 (9 to 38)	—	—	—	—
	Not white (n=32)	6 (2 to 16)	−.09 (−0.54 to 0.35)	.68	.01 (−0.43 to 0.45)	.96	17 (10 to 27)	−.06 (−0.42 to 0.29)	.74	.04 (−0.30 to 0.39)	.81	
**Educational qualification**											
	Pre-16 (reference; n=188)	4 (2 to 13)	—	—	—	—	15 (8 to 34)	—	—	—	—
	Post-16 (n=484)	6 (2 to 18)	.31 (0.10 to 0.52)	.004	.36 (0.15 to 0.57)	<.001	18 (9 to 41)	.12 (−0.05 to 0.28)	.18	.18 (0.02 to 0.16)	.03	
**Current smoker**											
	Yes (reference; n=165)	4 (2 to 16)	—	—	—	—	15 (8 to 29)	—	—	—	—
	No (n=507)	6 (2 to 18)	.20 (−0.02 to 0.42)	.08	.01 (−0.22 to 0.23)	.96	18 (9 to 39)	.16 (−0.01 to 0.34)	.07	−.02 (−0.19 to 0.16)	.84
Past week alcohol consumption (units)^e^		—	0 (0)	.570	0 (0)	.75	—	0 (0)	.47	0 (0)	.37
Alcohol use (AUDIT^f^ score)		—	−.01 (−0.02 to 0)	.19	−.01 (−0.02 to 0.01)	.42	—	0 (–0.01 to 0.01)	.64	0 (–0.01 to 0.01)	.94

^a^IQR: interquartile range.

^b^Adjusted for all sociodemographic variables, AUDIT score, and treatment group (unless otherwise specified).

^c^Unstandardized regression coefficient.

^d^Not applicable.

^e^Adjusted for all sociodemographic variables and treatment group (not AUDIT score).

^f^AUDIT: Alcohol Use Disorder Identification Test.

**Table 3:** The effect of user characteristics on measures of engagement (number of days used and % of screens viewed).

User characteristics		Days used, median (IQR^a^)	Unadjusted simple linear regression		Adjusted^b^ multiple regression		Screens viewed, % mean (SD)	Unadjusted simple linear regression		Adjusted^b^ multiple regression	
			B^c^ (95% CI)	*P* value	B (95% CI)	*P* value		B (95% CI)	*P* value	B (95% CI)	*P* value
Age (years)		—^d^	.02 (0.01 to 0.03)	<.001	.02 (0.02 to 0.03)	<.001	—	.25 (0.16 to 0.34)	<.001	.28 (0.19 to 0.38)	<.001
**Gender**											
	Male (reference; n=295)	4 (2 to 11)	—	—	—	—	37.7 (13.54)	—	—	—	—
	Female (n=377)	5 (2 to 13)	.12 (−0.05 to 0.30)	.16	.10 (−0.07 to 0.27)	.25	40.0 (13.54)	2.29 (0.27 to 4.32)	.03	1.99 (0.01 to 3.97)	.049	
**Employment status**											
	Unemployed (reference; n=91)	3 (1 to 11)	—	—	—	—	38.6 (13.97)	—	—	—	—
	Employed (n=581)	4 (2 to 13)	.16 (−0.09 to 0.41)	.22	.18 (−0.08 to 0.43)	.17	39.0 (13.22)	.37 (−2.58 to 3.32)	.80	.07 (−2.88 to 3.02)	.96
**Ethnicity**											
	White (reference; n=640)	4 (2 to 13)	—	—	—	—	39.1 (13.29)	—	—	—	—
	Not white (n=32)	4 (1 to 13)	−.13 (−0.54 to 0.27)	.52	−.04 (−0.44 to 0.36)	.83	36.9 (13.84)	−2.12 (−6.86 to 2.61)	.38	−1.48 (−6.16 to 3.19)	.53	
**Education**											
	Pre-16 years (reference; n=188)	3 (1 to 9)	—	—	—	—	36.7 (13.27)	—	—	—	—
	Post-16 years (n=484)	5 (2 to 14)	.23 (0.04 to 0.42)	.02	.28 (0.10 to 0.47)	.003	39.8 (13.24)	3.15 (0.91 to 5.38)	.006	4.04 (1.85 to 6.24)	<.001	
**Current smoker**											
	Yes (reference; n=165)	3 (1 to 12)	—	—	—	—	37.8 (12.72)	—	—	—	—
	No (n=507)	5 (2 to 13)	.20 (0 to 0.39)	.05	.02 (−0.02 to 0.01)	.85	39.4 (13.49)	1.57 (−0.77 to 3.91)	.19	−.05 (−2.40 to 2.30)	.97
Past week alcohol consumption (units)^e^		—	0 (–0.01 to 0)	.33	0 (0)	.42	—	0 (−0.04 to 0.04)	.98	0 (−0.03 to 0.04)	.87
Alcohol use (AUDIT^f^ score)		—	−.01 (−0.02 to 0)	.13	−.01 (−0.02 to 0.01)	.28	—	0 (−0.15 to 0.16)	.99	.02 (−0.13 to 0.17)	.82
										

^a^IQR: interquartile range.

^b^Adjusted for all sociodemographic variables, AUDIT score, and treatment group (unless otherwise specified).

^c^Unstandardized regression coefficient.

^d^Not applicable.

^e^Adjusted for all sociodemographic variables and treatment group (not AUDIT score).

^f^AUDIT: Alcohol Use Disorders Identification Test.

**Table 4:** The effect of user characteristics on response to follow up.

User characteristics		Completed follow up, n (%)	Unadjusted simple logistic regression		Adjusted^a^ multiple logistic regression	
			OR^b^ (95% CI)	*P* value	OR (95% CI)	*P* value
Age (years)		—^c^	1.04 (1.02-1.05)	<.001	1.04 (1.02-1.06)	<.001
**Gender**						
	Male (reference; n=295)	63 (21.4)	—	—	—	—
	Female (n=377)	116 (30.8)	1.64 (1.15-2.34)	.006	1.58 (1.09-2.29)	.02
**Employment status**						
	Unemployed (reference; n=91)	31 (34.1)	—	—	—	—
	Employed (n=581)	148 (25.5)	0.66 (0.42-1.07)	.09	0.66 (0.40-1.12)	.12
**Ethnicity**						
	White (reference; n=640)	172 (26.9)	—	—	—	—
	Not white (n=32)	7 (21.9)	0.76 (0.30-1.70)	.53	0.74 (0.28-1.73)	.51
**Educational qualification**						
	Pre-16 (reference; n=188)	36 (19.1)	—	—	—	—
	Post-16 (n=484)	143 (29.5)	1.77 (1.18-2.70)	.007	2.11 (1.38-3.29)	<.001
**Current smoker**						
	Yes (reference; n=165)	34 (20.6)	—	—	—	—
	No (n=507)	145 (28.6)	1.54 (1.02-2.38)	.045	1.23 (0.79-1.95)	.37
Past week alcohol consumption (units)^d^		—	1.00 (0.99-1.01)	.92	1.00 (1.00-1.01)	.56
Alcohol use (AUDIT^e^ score)		—	1.00 (0.97-1.02)	.72	1.00 (0.97-1.03)	.95

^a^Adjusted for all sociodemographic variables, AUDIT score, and treatment group (unless otherwise specified).

^b^OR: odds ratio.

^c^Not applicable.

^d^Adjusted for all sociodemographic variables and treatment group (not AUDIT score).

^e^AUDIT: Alcohol Use Disorder Identification Test.

### Associations Between User Characteristics and Extent of Alcohol Reduction at Follow Up Among Those Followed Up

Table 5 reports the unadjusted and adjusted linear regression models assessing the association between user characteristics and extent of alcohol reduction among those followed up. Past week alcohol consumption and AUDIT score (at baseline) were significantly positively associated with extent of alcohol reduction, with those having a higher past week alcohol consumption and greater AUDIT scores at baseline reducing their alcohol consumption to a greater extent at follow up. No sociodemographic user characteristics were significantly associated with extent of alcohol reduction among those followed up.

### Associations Between Measures of Engagement and Likelihood of Response to Follow Up

Table 6 reports the unadjusted and adjusted logistic regression models assessing the association between engagement and response to follow up. All engagement measures were significantly associated with likelihood of response to follow up, whereby greater engagement increased the likelihood of responding to follow up. This held true for the adjusted model when the known predictors of response to follow up were adjusted for.

### Associations Between Measures of Engagement and Extent of Alcohol Reduction at Follow Up Among Those Followed Up

Table 7 reports the unadjusted and adjusted linear regression models assessing the association between engagement and extent of alcohol reduction. No association between engagement and the extent of alcohol reduction was detected in any of the unadjusted models or the adjusted model.

### Sensitivity Analyses

Sensitivity analyses were conducted in which the engagement measures were dichotomized into high or low groups based on their median score (except for percentage of available screens viewed, which was dichotomized based on the mean score). The pattern of results remained the same.

**Table 5:** The effect of user characteristics on extent of alcohol reduction.

User characteristics		Mean (SD)	Unadjusted linear regression		Adjusted^a^ multiple regression	
			B^b^ (95% CI)	*P* value	B (95% CI)	*P* value
Age (years)		—^c^	−.13 (−0.44 to 0.18)	.42	−.11 (−19.71 to 27.33)	.53
**Gender**						
	Male (reference; n=63)	14.4 (26.25)	—	—	—	—
	Female (n=116)	14.2 (22.97)	−.20 (−7.67 to 7.26)	.96	.51 (−6.95 to 7.96)	.89
**Employment status**						
	Unemployed (reference; n=31)	10.9 (21.4)	—	—	—	—
	Employed (n=148)	15.0 (24.63)	4.05 (−5.35 to 13.45)	.40	5.07 (−4.73 to 14.89)	.31
**Ethnicity**						
	White (reference; n=172)	14.5 (24.28)	—	—	—	—
	Not white (n=7)	8.6 (19.91)	−5.92 (−24.29 to 12.45)	.53	−7.17 (−25.84 to 11.49)	.45
**Educational qualification**						
	Pre-16 (reference; n=36)	17.2 (26.49)	—	—	—	—
	Post-16 (n=143)	13.6 (23.51)	−3.58 (−12.46 to 5.30)	.43	−2.96 (−11.93 to 6.01)	.52
**Current smoker**							
	Yes (reference; n=34)	15.9 (25.42)	—	—	—	—
	No (n=145)	13.9 (23.86)	−1.97 (−11.05 to 7.11)	.67	.45 (−8.91 to 9.81)	.93
Past week alcohol consumption (units)^d^		—	.49 (0.37 to 0.61)	<.001	.49 (0.37 to 0.62)	<.001
Alcohol use (AUDIT^e^ score)		—	1.01 (0.46 to 1.55)	<.001	.98 (0.40 to 1.55)	<.001

^a^Adjusted for all sociodemographic variables, AUDIT score, and treatment group (unless otherwise specified).

^b^Unstandardized regression coefficient.

^c^Not applicable.

^d^Adjusted for all sociodemographic variables and treatment group (not AUDIT score).

^e^AUDIT: Alcohol Use Disorder Identification Test.

**Table 6:** The association between engagement and response to follow up.

Engagement measures		Statistics	Unadjusted simple logistic regression		Adjusted^a^ multiple logistic regression	
			OR^b^ (95% CI)	*P* value	OR (95% CI)	*P* value
**Sessions, median (IQR^c^)**						
	Did not respond (reference)	4 (2-11)	—^d^	—	—	—
	Responded	19 (8-32)	1.08 (1.06-1.10)	<.001	1.08 (1.06-1.09)	<.001
**Time on app, median (IQR)**						
	Did not respond (reference)	13.2 (7.4-26.0)	—	—	—	—
	Responded	35.1 (18.9-70.9)	1.02 (1.02-1.03)	<.001	1.02 (1.02-1.03)	<.001
**Days used, median (IQR)**						
	Did not respond (reference)	3 (1-8)	—	—	—	—
	Responded	14 (6-24)	1.14 (1.11-1.17)	<.001	1.13 (1.11- 1.16)	<.001
**Percentage of available screens viewed, mean (SD)**						
	Did not respond (reference)	35.6 (12.14)	—	—	—	—
	Responded	48.3 (11.92)	1.09 (1.07-1.11)	<.001	1.09 (1.07-1.11)	<.001

^a^Adjusted for treatment group, age, gender, and education group (as significant predictors of response to follow up).

^b^OR: odds ratio.

^c^IQR: interquartile range.

^d^Not applicable.

**Table 7:** The association between engagement and extent of alcohol reduction.

Engagement measures	Unadjusted linear regression		Adjusted^a^ multiple linear regression	
	B^b^ (95% CI)	*P* value	B (95% CI)	*P* value
Sessions	−.16 (−0.37 to 0.05)	.13	−.14 (−0.34 to 0.07)	.19
Time on app	−.06 (−0.13 to 0.01)	.08	−.06 (−0.13 to 0.01)	.07
Days used	−.18 (−0.57 to 0.21)	.37	−.10 (−0.48 to 0.28)	.61
Available screens viewed	−.07 (−0.37 to 0.23)	.66	−.09 (−0.39 to 0.22)	.58

^a^Adjusted for treatment group and baseline AUDIT score (as a significant predictor of extent of alcohol reduction).

^b^Unstandardized regression coefficient.

## Discussion

### Summary of Principal Findings

#### Engagement and Response to Follow Up

Users who were older and had post-16 educational qualifications engaged with the Drink Less app to a greater extent, which was indicated by number of sessions, time on app, number of days used, and percentage of available screens viewed. Female users viewed a significantly greater percentage of available screens compared with male users. Users who were older, female, and had post-16 educational qualifications were also significantly more likely to respond to follow up. In line with previous literature from Web-based alcohol interventions [16,19-21], users who were female, older, and with post-16 educational qualifications engaged to a greater extent and were more likely to respond to follow up. This suggests that there are similarities in the user characteristics that are predictors of engagement and response to follow up between app- and Web-based alcohol interventions. However, this study found that gender was only associated with the percentage of available screens viewed (ie, depth of use) and was not associated with more typical measures of engagement such as amount or frequency of use.

All 4 measures of engagement were positively associated with the likelihood of responding to follow up, and this association remained when adjusting for the user characteristics that were significant predictors of response to follow up, which replicated previous findings [15,16]. There was no evidence that drinking characteristics were associated with engagement or the likelihood of response to follow up. This contradicts previous literature that has found that drinking characteristics are positively associated with engagement and negatively associated with response to follow up in Web-based alcohol interventions. A possible explanation could be the delivery modality (ie, app vs website) [33] or the intervention content. For example, Drink Less relies on a quick on-boarding process and involves no contact with health care professionals, which may have resulted in users feeling less stigmatized and more likely to return irrespective of their drinking status. Alternatively, the content of the Drink Less app (eg, a game, a drinking diary) might have been sufficiently rewarding to promote engagement irrespective of drinking. Due to the lack of existing evidence relating to app-based interventions, it is not possible to draw any firm conclusions as to what may have caused the difference between the findings from this study and the association found between drinking characteristics and engagement and response to follow up in Web-based alcohol interventions.

#### Extent of Alcohol Reduction

Past week alcohol consumption and AUDIT score were both positively associated with the extent of alcohol reduction among those followed up. None of the sociodemographic characteristics were associated with the extent of alcohol reduction. There has been a lack of research on the predictors of alcohol reduction in the general population, particularly in app-based interventions. Our study found that drinking characteristics were positively associated with the extent of alcohol reduction. This is likely explained by regression to the mean, as measurements that differ substantially from the true mean tend to be followed by measurements closer to the true mean [40]. Further research is needed to elucidate whether this observation is driven by regression to the mean or whether app-based interventions tend to be more effective for people with higher levels of alcohol consumption. This could be tested with a confirmatory RCT comparing an optimized version of the app with a no-treatment control group to examine whether the same patterns of associations (ie, between drinking characteristics and alcohol reduction) are also observed in the control group.

No associations were detected between the engagement measures and the extent of alcohol reduction among those followed up, and these results were robust to a sensitivity analysis with the engagement measures as dichotomous variables. This means that we were not able to determine whether there is a threshold level of engagement with the app that would achieve users’ intended reduction in alcohol consumption. These findings conflict previous findings of a positive association between engagement measures and successful behavior change [30-32,41]. This difference may be because of a different methodology used; in this study, we only analyzed the subsample of participants who completed the follow-up questionnaire, whereas many studies use an intent-to-treat approach [30,31,41]. Another possibility is that people use the Drink Less app in different ways because of the complexity of the app’s design, meaning that threshold level of engagement differs across users. The strong association reported in previous studies might be driven by people who are unsuccessful in their behavior change, disengage with the app, and then do not complete the follow up. Nevertheless, a dose-response relationship between engagement and alcohol reduction would still be expected among those users who responded to follow up. The finding that engagement is not related to successful behavior change through a dose-response function is consistent with the findings from the factorial RCT that certain combinations of Drink Less modules were more effective than others [7]. An unplanned analysis found that greater engagement with the app mediated the effect of the self-monitoring module on reduction in AUDIT score for those users who received the combination of self-monitoring and action planning [42]. This mediation effect suggests that an engagement dose-response effect may depend on the intervention module. It is also possible that the threshold level of engagement for the intended outcomes of Drink Less was relatively low for all users (compared with other DBCIs), and a ceiling effect may have played a role in not detecting an overall dose-response effect between the extent of engagement and alcohol reduction.

### Implications

Tailored strategies for younger male users with lower educational qualifications, who tend to have lower levels of engagement and response to follow up, could be codeveloped with these users to improve engagement and response to follow up. Users who were older and had post-16 educational qualifications engaged with the app to a greater extent in terms of number of sessions, time spent on the app, the number of days it was used for, and the depth of their use. The app was not designed for a specific age group (other than the adult population) and involved user testing with participants from disadvantaged groups who typically have poorer Web-based literacy to ensure it was usable and acceptable to these groups [34]. A possible explanation for the difference in engagement based on the user’s age is that different age groups might differ in the ways in which they tend to engage with apps more generally (eg, younger users being less willing to spend a lot of time on apps). Future research should use the data available from alcohol reduction apps to investigate whether there are different user typologies and if these are categorized by age.

The finding that engagement measures were not associated with the extent of alcohol reduction suggests that engagement measures should not be used as a proxy for behavior change and that greater levels of engagement are not necessarily required to achieve a desired change in behavior. Therefore, tailored strategies for improving engagement and response to follow up will not necessarily result in the desired behavior change.

### Strengths and Limitations

To our knowledge, this is the first study to investigate the predictors of engagement, response to follow up, and extent of alcohol reduction in an app-based intervention. Drink Less is freely available in the UK iTunes App Store and users were not directly recruited for a trial; instead, they downloaded the app and were then recruited to the trial. Therefore, this sample has high ecological validity and represents the real-world situation for most users of behavior change apps. This study had a modest sample size and could be repeated with a larger sample to assess whether the findings are replicable. A limitation of this study is that the measures of engagement used were summative and, therefore, could not be used to assess more specific patterns of engagement (eg, the order in which users engaged with the app’s different components), which future research should look to investigate.

### Conclusions

Users of an alcohol reduction app who were older and had post-16 educational qualifications engaged to a greater extent. These characteristics and being female predicted users being more likely to respond to a follow-up questionnaire 1 month later. Higher baseline levels of alcohol consumption were predictive of a greater extent of alcohol reduction, but were not predictive of engagement or response to follow up. Engagement measures were significantly associated with response to follow up, in line with the law of attrition. Engagement measures were not associated with the extent of alcohol reduction, which suggests that there is no dose-response effect of the Drink Less app.

## Abbreviations

AUDIT: Alcohol Use Disorders Identification Test
AUDIT-C: Alcohol Use Disorders Identification Test-Consumption
CRUK: Cancer Research UK
DBCI: digital behavior change intervention
IQR: interquartile range
NIHR: National Institute for Health Research
OR: odds ratio
SPHR: School for Public Health Research
RCT: randomized controlled trial
UKCTAS: UK Centre for Tobacco and Alcohol Studies
